# Rehabilitation research in spinal muscular atrophy: a call to action

**DOI:** 10.1177/22143602251364945

**Published:** 2025-08-25

**Authors:** Charlotte Lilien, Leslie Nelson, Lisa Edel, Danielle Forrest, Timothy Estilow, Katlyn E McGrattan, Tina Duong, Giorgia Coratti

**Affiliations:** 1 STRONG MDUK Oxford Neuromuscular Centre, Department of Paediatrics, 121582 University of Oxford, Oxford, UK; 2 Department of Physical Therapy, 685149 University of Texas Southwestern Medical Center, Dallas, TX, USA; 3 4956 Great Ormond Street Hospital, London, UK; 4 Institute of Child Health, 11700 University College London, London, UK; 5 Department of Occupational Therapy, 6567 The Children's Hospital of Philadelphia, Philadelphia, Pennsylvania, USA; 6 Department of Speech Language and Hearing Science, 5635 University of Minnesota, Minneapolis, MN, USA; 7 John W. Day Lab, Department of Neurology and Neurological Sciences, 214563 University of Stanford, Stanford, USA; 8 Pediatric Neurology, 60234 Catholic University of Sacred Heart, Rome, Italy; 9 Centro Clinico Nemo, 18654 Fondazione Policlinico Universitario Agostino Gemelli IRCCS, Rome, Italy

**Keywords:** SMA, rehabilitation, research, barriers, challenges

## Abstract

**Design:**

The landscape of spinal muscular atrophy drastically changed following the introduction of disease-modifying therapies, emphasizing the need for comprehensive rehabilitation strategies to maximize functional outcomes. Our aim is to identify the barriers faced by healthcare professionals in conducting rehabilitation research for spinal muscular atrophy and suggest potential solutions to increase evidence-based care.

**Methods:**

We performed a narrative review of rehabilitation intervention studies with a quality assessment focusing on the research characteristics of each study and an international survey questioning healthcare professionals on their major areas of difficulty in conducting interventional studies.

**Results:**

The review highlighted a predominantly low to unacceptable quality of the 36 retained studies across four healthcare professional domains responsible for rehabilitation: physiotherapy/physical therapy (18), occupational therapy (4), respiratory therapy (10), and speech and language therapy/speech language pathology (4).

The survey was completed by 204 health care providers from 35 countries. Funding was found to be the most significant barrier to rehabilitation research, followed by study design and recruitment challenges.

**Conclusion:**

Our findings highlight the urgent need for randomized controlled trials and standardized methodologies to develop robust, evidence-based multi-disciplinary rehabilitation strategies that better support individuals with spinal muscular atrophy in achieving optimal functional outcomes in the era of disease-modifying treatments.

## Introduction

With an incidence of 1:14.848 births, spinal muscular atrophy (SMA) is a progressive muscle wasting condition.^1^ SMA was originally classified based on the highest motor milestone achieved and the age at symptom onset. This ranged from the most severe and often fatal cases (type I), presenting with symptoms before 6 months of age, to patients who never attain the ability to walk (type II), those who initially walk but later lose this ability (type III), and those with milder symptoms appearing in adulthood (type IV).^2,3^ Since 2016, three disease-modifying therapy (DMT) for SMA have been approved by the Food and Drug Administration and European Medical Agency.^4–6^ These therapies, alongside early treatment^7^ and proactive care as per the Standards of Care (SoC) guidelines^8,9^ have transformed the SMA landscape, presenting new challenges for clinical care teams.^10^ Longer survival rate did not necessarily ameliorate symptoms of the disease.^11^ Despite real world evidence associated with stabilization or even functional/structural improvements of the disease and increased patient's quality of life, there continues to be functional limitations and disability associated with bulbar weakness, muscle imbalances, contractures and scoliosis that are continued gaps in knowledge that may be impacted by allied health professionals.^12–14^

The resulting shifts in SMA phenotypes given by DMTs have spurred the scientific community to reconsider current classifications and care recommendations,^15,16^ with adapted best practices but yet no definitive new SoC to reflect this new era.^17,18^ While considered the gold standard to implement new techniques in care, randomized controlled trials are particularly limited in rehabilitation research, the neuromuscular field, and especially in SMA.^19–23^ Yet, alternative approaches such as real-world evidence, observational studies, and pragmatic trials also play a critical role in advancing care, particularly when traditional randomized controlled trials are not feasible.

The aim of this study is twofold: (1) to understand the evidence-based research and gaps that currently exists in the field of rehabilitation for SMA, and (2) to gather insights from rehabilitation professionals regarding the barriers preventing the conduction of interventional research in SMA rehabilitation. Identification of research gaps and research barriers is key to addressing solutions to increase quality of research in rehabilitation medicine that may benefit individuals with SMA in the DMT era. The study does not aim to review the rehabilitation methods or their effectiveness but rather to investigate and ultimately address the gaps in rehabilitation research in SMA. This understanding will be required to support clinicians contributing to understanding the complementary impacts of rehabilitation with DMTs.

## Methods

This project is divided into two separate phases:

### Phase 1: narrative review and quality assessment

Healthcare professional (HCP) domains included within this research (physiotherapy/physical therapy,^24^ respiratory therapy,^25^ speech and language therapy/pathology,^26^ occupational therapy^27^) refer to clinical needs highlighted in SMA SoC.^8,9^ To assess the barriers encountered by HCPs in SMA-related research, a narrative review of existing literature was deemed appropriate. Indeed, a narrative review offers a general summary that includes interpretation, critical analysis, and context informed by the survey findings (Phase 2) and expert opinion of the authors.^28^ Relevant literature was identified on Medline (PubMed) and specific keywords and Mesh Terms can be found in Supplementary 1. The review only included original, full-text clinical research articles focused on interventional studies (randomized controlled trials, non-randomized controlled trials, cohort studies, case studies) published in English after 1st of January 2016, aligning with the approval of the first DMT in October 2016. A decision-flowchart helped to identify and select the appropriate publications across the different authors (Supplementary 2). The narrative review was conducted from April to June 2024 to inform the survey and was subsequently extended through February 2025 to capture as much literature as possible. To highlight the methodological aspects of research conducted by HCPs, which could have been impacted and or limited by barriers, a quality assessment has been conducted for each publication, although usually not required for narrative reviews. The quality and design of each study were assessed and scored using part B of the Critical Appraisal Tool for Experimental Intervention Studies (APTA CAT-EI).^29^ Two independent reviewers applied the tool to each study to ensure consistency and reduce potential biases in the evaluation process. In case of disagreements, a third reviewer was considered. Each item of the tool was assigned with a score (0 = no/NA to 1 = yes). Since criteria can be set according to needs, levels of overall quality were attributed depending on the score and assessed according to authors experience: High (8<) - Acceptable (6–8) - Low (3–5) - Unacceptable (0–2).^30^ Ultimately, the narrative review and the quality assessment supported the confirmation/completion of the barriers used for the survey (Phase 2) initially identified by the authors.

### Phase 2: the survey

Following the initial findings of the narrative review (April - June 2024), a web-based survey was created to understand and correlate the review findings into context with real world barriers to quality research in HCPs in SMA (Supplementary 3). The survey items were derived from key barriers identified during the narrative review process and refined with input from experts in SMA care to ensure relevance and clarity. Key authors identified nine potential research barriers and asked respondents to rank them. A pilot test was conducted with a small group of HCPs (authors of this publication) to validate the survey's content including the barriers and structure. Based on the pilot test feedback, minor adjustments were made to enhance the clarity of the questions and ensure ease of completion. The survey was distributed internationally by the authors via professional networks (patient-advocacy groups, industry partners, national and international networks of colleagues) utilizing a non-probability convenience sampling approach. Data were collected cross-sectionally from June to August 2024. To ensure the anonymity and confidentiality of survey respondents, the survey was conducted through Jotform (https://www.jotform.com/), which implements several mechanisms to protect data. Respondents were not asked to provide personally identifiable information unless required for the study purpose, maintaining their anonymity throughout the process. The platform uses SSL encryption to secure all data transmitted, ensuring that responses are protected from unauthorized access. Additionally, Jotform tracks IP addresses and uses cookies to prevent multiple submissions from the same user, which helps maintain data integrity and avoid multiple participations. Access to the collected data was restricted to key authors of this manuscript, with strict controls in place to safeguard the privacy of respondents’ information. HCPs were requested to provide general demographic details on the country of practice, role, professional degree, and experience with SMA and to rank barriers provided by the authors from 1–9 (the most important - the least important).

The survey was developed with the primary aim of capturing which barriers were perceived as relevant by stakeholders in rehabilitation-related research. It was designed as a brief, pragmatic tool to facilitate broad engagement and rapid insight-gathering. The development was informed by a narrative review that highlighted common themes and barriers identified in previous studies by the authors. These were grouped into key domains and presented in a simplified format to encourage participation and minimize respondent burden.

The survey consisted of a demographic section, two barrier-related questions (one structured with multiple sub-items and one open-ended to provide missing barriers not disclosed in the questionnaire). Given the exploratory nature of the study, a single multi-factorial question was used to prompt initial reactions and gather a wide range of inputs. No ethical approval was required since no vulnerable subjects were targeted, and the questions were not leading to any physical or psychological harm.^31^

Statistical analysis:

For each participant the response ranked as 1 is the most important and the response ranked as 9 is the least important, numeric values were assigned to these responses.

With the numeric values in place, the average rating for each research challenge was calculated. This involved grouping the data by the specific research challenge and computing the mean rating assigned by all respondents.

Finally, the challenges based on their average ratings were ranked. The challenges with lower average ratings are considered more important, as they were assigned higher rankings (i.e., closer to 1).

Analysis was conducted stratified by geographical area, HCP domain, and interest/involvement/opportunity in clinical research in SMA. This will help to understand whether these factors influence the degree of agreement in rankings and correlations observed, thereby providing insights into potential regional or professional variations in perspectives or experiences related to SMA clinical research. By examining these subgroups separately, we sought to identify patterns of concordance or divergence that may inform targeted strategies for collaboration, education, or resource allocation within the SMA research community.^32–40^

To assess the overall concordance of rankings across groups, we employed Kendall's coefficient of concordance (Kendall's W). Values of Kendall's W range from 0 (no agreement) to 1 (complete agreement). Statistical significance of Kendall's W was evaluated using the associated chi-square (χ²) test.

Following the identification of significant overall concordance, we conducted pairwise correlation analyses between regions using Kendall's tau (τ), a non-parametric measure of rank correlation that assesses the strength and direction of association between two sets of rankings. Kendall's tau values range from −1 (complete disagreement) to +1 (complete agreement).

Given the multiple pairwise comparisons performed, we applied a multiple testing correction to control for the family-wise error rate. Specifically, the Benjamini-Hochberg procedure was used to adjust p-values derived from the Kendall's tau tests, balancing type I error control with statistical power. Only pairwise correlations with an adjusted p-value below the significance threshold (α = 0.05) were considered statistically significant.

Responses to the open-ended question were analysed using basic thematic coding. Two independent reviewers categorized the responses into emergent themes based on content similarity. Discrepancies were resolved through discussion.

## Results

### Phase 1: literature review and quality assessment

The literature search identified publications focusing on physiotherapy/physical therapy (n = 148), respiratory therapy (n = 92), speech and language therapy/pathology (n = 59) and occupational therapy (n = 5). Following the decision-tree, a total of 36 eligible papers distributed unequally across each HCP domain were retained for review and quality assessment (Table 1).

**Table 1.** Narrative review and quality assessment (APTA-CAT-EI).

Literature review								Quality assessment: APTA CAT-EI
Healthcare professional domain	First Author, year	Study design: pilot study, case study, case series, cross-sectional, prospective/retrospective	Study objective	Study duration per participant	Number of participants for results presentation and population (adult and/or pediatrics)	Treatment with any disease-modifying therapy	Presence of control group	Quality: High (8<) - Acceptable (6–8) - Low (3–5) - Unacceptable (0–2)
Physiotherapy/Physical therapy	Gamze Bora, 2018	Pilot study, prospective	To evaluate the effects of arm cycling exercise on the physical function of SMA Type II patients and to link exercise-induced benefits with SMN, IGF1, and IGF-binding protein 3 (IGFBP3) levels.	12 weeks	N = 5; paediatric	Not specified	No	Acceptable
Physiotherapy/Physical therapy	Habets, 2022	Cross sectional, intervention with gender matched controls	Evaluate for SMA 3 and 4 and age matched controls to perform upper body exercise test to see if changes in oxygen consumption, strength, motor unit recruitment and capillary recruitment with max performance exercise	Cross sectional, 1 session	N = 15 SMA with n = 3 type a3; n = 8 type 3b, n = 1 type 4 N = 15 aged and gender matchec controls	No	Yes	Acceptable
Physiotherapy/Physical therapy	Immarino et al. 2024	Prospective; case series	Feasibility and utility of in-home body weight support harness system use in young children treated for spinal muscular atrophy	6 months	N = 32; paediatric	Yes	nN	Acceptable
Physiotherapy/Physical therapy	Nishikawa et al. 2021	Case study	To quantify the effects of Hybrid Assistive Limb (HAL) gait training on spatial muscle activity patterns in a patient with SMA type III using multi-channel surface electromyography (SEMG)	1 session	N = 1; adult	Not specified	No	Low
Physiotherapy/Physical therapy	Bulut et al. 2019	Case study; prospective	To study the effect of ergometer and aquatherapy in a child with spinal muscular atrophy type II	Ergometer, 12 weeks; Aquatherapy,12 weeks	N = 1; paediatric	Not specified	No	Low
Physiotherapy/Physical therapy	Stark et al. 2018	Retrospective; observational (intervention implemented in practice)	To study the effect of a vibration-assisted home training program in children with spinal muscular atrophy	12 months	Baseline, N = 38; Month 12, N = 15; paediatric	No	No	Acceptable
Physiotherapy/Physical therapy	Nakajima et al. 2021	Multicentre; open-label; controlled; crossover	To evaluate the efficacy and safety of cybernic treatment with a wearable cyborg Hybrid Assistive Limb (HAL, Lower Limb Type) in improving the ambulatory function in patients with slowly progressive rare neuromuscular diseases	8.5 months	N = 30; SMA, N = 5; adults	No	Yes, crossover study	High
Physiotherapy/Physical therapy	Gobbo et al. 2019	Case report; prospective	Exercise Combined with Electrotherapy Enhances Motor Function in an Adolescent with Spinal Muscular Atrophy Type III	18 months	N = 1; paediatric	Not specified	No	Low
Physiotherapy/Physical therapy	Mirea,2022	Retrospective; observational study (intervention implemented in practice)	To objectify better outcome in SMA patients that receive nusinersen treatment and physical therapy	12 months	N = 39 (treated); N = 16 (untreated), paediatric	Yes	Yes	Acceptable
Physiotherapy/Physical therapy mixed with respiratory therapy (comprised within the Physiotherapy section for this review)	Leung Yu, 2023	Prospective case series	Feasibility, safety and efficacy of a 16-week integrated respiratory and motor telerehabilitation program for a pilot cohort of stable patients with hereditary pediatric NMDs.	16-week	N = 8; N = 4 SMA, paediatric and adult	Yes, some	No	Acceptable
Physiotherapy/Physical therapy	Cumplido-Trasmonte, 2022	Case series;	Effects before and after using the “ATLAS 2030” exoskeleton in a population of children with SMA type II (1) to measure increase or decrease in the maximal isometric strength of the muscles groups responsible for lower extremity movement, (2) to assess the changes in ROM of the hips, knees and ankles.	8-weeks	N = 3; paediatric	Yes	No	Low
Physiotherapy/Physical therapy	Moshonkina, 2024	Case series	tSCS could activate intact motor neurons and motor neurons that were restored after treatment with pathogenic drugs, leading to a slower decline in motor activity and the development of motor skills	2-week	N = 37, adult; N = 1 type 2 SMA; N = 6 type 3 SMA	Yes	No	Low
Physiotherapy/Physical therapy	Lammers,2024	Case series	To describe the feasibility and effect of caregiver-mediated exercise training using a novel Therapeutic Play Gym in 3 neonatal intensive care unit (NICU) graduates with rare neuromuscular diseases.	6 months	N = 1; paediatric	No	No	Low
Physiotherapy/Physical therapy	Taylor, 2024	Case report	To support client-centred goal achievement and rehabilitation for children with SMA	10 weeks	N = 1; paedaitric	Not specified	No	Unacceptable
Physiotherapy/Physical therapy	Novikov, 2023	Case Series	Stimulation can activate intact motor neurons and motor neurons that have been restored after nusinersen treatment, resulting in a deceleration in the decline in motor activity and the development of motor skills.	tSCS during physical therapy 30–40 min per day in the course of 10–14 days	N = 5; paediatric	Yes	No	Low
Physiotherapy/Physical therapy	Ahmed, 2024	Case Report	Overview of the therapeutic exercise rehabilitation program used for a child with type 2 SMA in an inclusive learning environment	12 months	N = 1; paediatric	Not specified	No	Low
Physiotherapy/Physical therapy	Kanner, 2025	Case report	To report on therapeutic scoliosis-specific exercises (PSSE) for a child with SMA who had spinal fusion.	Pre-op: 15 sessions over 6 months Post-op (20 months later): 9 sessions over 4 months	N = 1; paediatric	Yes	No	Low
Physiotherapy/Physical therapy	Prat-Ortega, 2025	Case series	To report first-in-human study of epidural spinal cord stimulation in individuals with spinal muscular atrophy	4 weeks, 2 h per day	N = 3; adult	Yes, 2/3	No	Low
Respiratory therapy	Bertelli, 2017	Case report	Use of Free Aspire in patients with ineffective cough and impaired removal of secretions	Unclear - a few days	N = 1 paediatric	No	No	Unacceptable
Respiratory therapy	Chen, 2016	Case reports	Early implementation of combined NIV/MIE	Unclear	N = 2	No	No	Low
Respiratory therapy	DPT	Prospective	evaluate the effects of LTNPPV on sleep architecture, and microstructure (by means of CAP analysis) of children with SMA2 and to assess their residual difference from age- and sex-matched controls, after this intervention	No longitudinal follow up	N = 9 + N = 15 aged matched controls	No	Yes	Low
Respiratory therapy	Panitch, 2017	Expert opinion	Should we be using trache for SMA1	N/A	N/A	No	N/A	Unacceptable
Respiratory therapy	Bach, 2017	Expert opinion	Is NIV appropriate in SMA1	N/A	N/A	No	N/A	Unacceptable
Respiratory therapy	Senel, 2020	Prospective; case series	Evaluate the possible beneficial effects of the non-invasive mechanical ventilation on cardiac autonomic dysfunction caused by OSAS in patients with SMA	N/A	N = 12 6 SMA 6 Control	No	Yes	Unacceptable
Respiratory therapy	Bach, 2019	Case reports	Mechanical In-exsufflation-Expiratory Flows as Indication for Tracheostomy Tube Decannulation	N/A	N = 2	No	N/A	Unacceptable
Respiratory therapy	Suh, 2018	Retrospective; observational study (intervention implemented in practice)	Application of NIV in different NMD patients	5 years	N = 180	No	N/A	Unacceptable
Respiratory therapy	Chatwin, 2020	Retrospective; observational study (intervention implemented in practice)	Adherence to treatment recommendations, safety, settings, and discontinuation of Mechanical insufflation-exsufflation	N/A	N = 181	Not specified	N/A	Acceptable
Respiratory therapy	Veldhoen, 2022	Case series	Studied the effect of either air stacking and MI-E on lung function tests up to 2 h after ACT in patients with NMDs not naıve to treatment	2 h	N = 67	Not specified	N/A	Unacceptable
Occupational therapy	Rice et al, 2019	Repeated Measures Study	To examine the influence of use of the anterior tilt-in-space power seat function on performance of functional activities, physical health, and user satisfaction on among power wheelchair users	2 weeks 4 days	N = 10 overall, but only 3 SMA	Not specified	Subjects served as their own control at baseline and then f/u was with the intervention (anterior tilt of w/c)	Acceptable
Occupational therapy	Varela Aldas, et al 2024	Case report	Analyze the usability and performance of an assistive system based on the IoT when is evaluated by a child patient with spinal muscular atrophy type 1 (SMA-I)	30 days	N = 1	Yes	No	Low
Occupational therapy	Vogel, et al 2018	Proof of concept study with case control	The goal of this study was to design an sEMG-based interface which allows people with severe muscular atrophy to continuously control assistive devices in three degrees of freedom	4 sessions over 4 days	N = 2	Not specified	Yes, but control had previous exposure to intervention and was considered an “expert user”	Low
Occupational therapy	Janssen, 2021	Case series	Yumen Arm will improve upper extremity function and reduce fatiguability in persons with DMD and SMA	1 session	N = 6; N = 1 SMA, paediatric; N = 2, SMA, adult	Not Specified	No	Low
Speech and language therapy/ pathology	Chilińska-Pułkowska, 2021	Case report	Report management of a patient with SMA 1	2 years	N = 1 Sympomatic SMA Pediatric	Yes	No	Low
Speech and language therapy/ pathology	Okamoto, 2024	Case report, prospective	Elucidates that a patient with SMA could acquire vacuum swallowing with proper instructions	43 days	1 67 yo male	Not specified	no	Low
Speech and language therapy/ pathology	Morris, et al 2020	Retrospective Case Series	Determine whether the TheraBite would improve AROM or PROM of the temporomandibular joint.	Home program consisting of hourly stretches for 7 months	N = 3 NM, only 1 of them SMA	No	No	Low
Speech and language therapy/ pathology	McGrattan, et al 2024	Retrospective Case Series	Determine if thickened liquids improved swallowing during a VFSS exam	N/A	N = 37	No	Yes, their own control	Low

Key to table: Identified papers meeting the selection criteria. Spinal muscular atrophy (SMA), years old (yo). Treatment with any disease-modifying therapy considered as “No”, when cohort of patients was before 2016 but paper published after 2016 and “Not specified” when not clearly stated. Cohorts of patients aged below 18 years of age were considered as paediatric. APTA Critical Appraisal Tool for Experimental Intervention Studies: B. Quality Evaluation-Overall Design. ACT: Airway clearance techniques; AROM: Active range of motion; CAP: Cyclic alternating pattern; DMD: Duchenne muscular dystrophy; IGF: Insulin-like growth factor; MI-E: Mechanical insufflation–exsufflation; NIV: Non invasive ventialtion; NMD: Neuromuscular diseases; OSAS: Obstructive sleep apnea syndrome; OTD: Doctor of Occupational Therapy; PROM: Passive range of motion; ROM: Range of motion; SMN: Survival motor neuron; SSL: Secure sockets layer; ATLAS: Commercial name.

Details of the quality assessment including items and scores are available in Supplementary 4. The articles selected for analysis were examined from a research methodology perspective (e.g., presence of control groups, randomization, sampling, etc.) based on the APTA CAT-EI criteria. The effectiveness or utility of the studies was not assessed, as this is not within the scope of the study's objectives.

**Physiotherapy/Physical therapy:** Amongst 18 retained publications, 13 were case reports/studies. The quality of the papers was found to be high (n = 1), acceptable (n = 6), low (n = 10) and unacceptable (n = 1).

**Occupational therapy:**​​ Amongst the 4 retained publications, 3 were case series. The quality of the papers was found to be acceptable (n = 1) and low (n = 3).

**Respiratory therapy:** Amongst the 10 retained publications, 5 were case reports and 5 were expert opinion. The quality of the papers was found to be acceptable (n = 1), low (n = 2) and unacceptable (n = 7).

**Speech and language therapy/Speech language pathologist**: Amongst the 4 retained publications, all were case reports. This quality of the papers was found to be low (n = 4).

The narrative review supported the identification and confirmation of common barriers faced in the research studies such as small samples (case reports and case series), short duration (most studies were from one session to less than 12 months), interventions addressing multiple interventions or constructs, retrospective data collection and single site studies.

The only study amongst the 36 papers which scored with a high quality focused on physiotherapy with multiple centres, a control group and with 8.5 months follow-up. Only 10 of the 36 papers were conducted in pharmacologically treated patients: physiotherapy/physical therapy (8), occupational therapy (1), respiratory therapy (0), and speech and language therapy/speech language pathologist (1).

### Phase 2: the survey

A total of 204 HCPs actively involved in the care or research of SMA patients completed the survey. The respondents’ geographical distribution includes countries from higher income (e.g., Belgium and United Kingdom) to upper middle (e.g., Turkey and Brazil) and lower middle countries (e.g., Egypt and Tunisia). Since the survey was sent across networks for distribution the response rate could not be quantified by the authors. As it is commonly accepted that collecting over 100 surveys provides a sufficient basis for exploratory analyses, we ensured that our dataset included >100 responses to maintain the study's reliability and relevance.^41^ The country with the largest number of respondents was the UK, which contributed significantly to the overall sample, while the region with the fewest respondents was Australasia. No missing data were identified. Table 2 outlines the key characteristics of the 204 respondents, including their professional background, highest qualification, involvement in clinical research, and other relevant details. Countries have been pooled to have at least 4 participants responding by geographical area.

**Table 2.** Characteristics of survey respondents.

		Africa (N = 11)	Australasia (N = 4)	Canada (N = 12)	East EU (N = 9)	South EU (N = 43)	North EU (N = 18)	Singapore (N = 11)	South America (N = 9)	UK (N = 49)	USA (N = 38)	Overall (N = 204)
HCP domain	Physiotherapy	10 (90.9%)	2 (50.0%)	10 (83.3%)	7 (77.8%)	22 (51.2%)	15 (83.3%)	9 (81.8%)	4 (44.4%)	40 (81.6%)	25 (65.8%)	144 (70.6%)
	Speech & language therapy	1 (9.1%)	1 (25.0%)	-	2 (22.2%)	2 (4.7%)	-	2 (18.2%)	2 (22.2%)	-	4 (10.5%)	14 (6.9%)
	Occupational therapy	-	1 (25.0%)	2 (16.7%)	-	-	2 (11.1%)	-	2 (22.2%)	3 (6.1%)	9 (23.7%)	19 (9.3%)
	TNPEE	-	-	-	-	13 (30.2%)	0 (0%)	-	-	-	-	13 (6.4%)
	Respiratory therapy	-	-	-	-	6 (14.0%)	1 (5.6%)	-	1 (11.1%)	6 (12.2%)	-	14 (6.9%)
Highest qualification	BsC	5 (45.5%)	1 (25.0%)	8 (66.7%)	2 (22.2%)	23 (53.5%)	1 (5.6%)	9 (81.8%)	0 (0%)	31 (63.3%)	2 (5.3%)	82 (40.2%)
	DPT	1 (9.1%)	-	-	1 (11.1%)	1 (2.3%)	-	-	2 (22.2%)	-	19 (50.0%)	24 (11.8%)
	Msc	2 (18.2%)	2 (50.0%)	4 (33.3%)	2 (22.2%)	15 (34.9%)	10 (55.6%)	2 (18.2%)	4 (44.4%)	14 (28.6%)	11 (28.9%)	66 (32.4%)
	PhD	1 (9.1%)	1 (25.0%)	-	1 (11.1%)	2 (4.7%)	2 (11.1%)	-	1 (11.1%)	-	4 (10.5%)	12 (5.9%)
	Professional diploma	2 (18.2%)	-	-	3 (33.3%)	2 (4.7%)	5 (27.8%)	-	2 (22.2%)	3 (6.1%)	-	17 (8.3%)
	Postgraduate Diploma	-	-	-	-	-	-	-	-	1 (2.0%)	-	1 (0.5%)
	OTD	-	-	-	-	-	-	-	-	-	2 (5.3%)	2 (1.0%)
Involvement	No	4 (36.4%)	1 (25.0%)	5 (41.7%)	4 (44.4%)	13 (30.2%)	3 (16.7%)	9 (81.8%)	4 (44.4%)	18 (36.7%)	7 (18.4%)	68 (33.3%)
Yes	7 (63.6%)	3 (75.0%)	7 (58.3%)	5 (55.6%)	30 (69.8%)	15 (83.3%)	2 (18.2%)	5 (55.6%)	31 (63.3%)	31 (81.6%)	136 (66.7%)
Interest	Yes	11 (100%)	4 (100%)	7 (58.3%)	9 (100%)	37 (86.0%)	18 (100%)	4 (36.4%)	7 (77.8%)	44 (89.8%)	37 (97.4%)	178 (87.3%)
No	-	-	5 (41.7%)	-	6 (14.0%)	-	7 (63.6%)	2 (22.2%)	5 (10.2%)	1 (2.6%)	26 (12.7%)
Opportunity	No	4 (36.4%)	1 (25.0%)	5 (41.7%)	2 (22.2%)	9 (20.9%)	4 (22.2%)	7 (63.6%)	1 (11.1%)	21 (42.9%)	8 (21.1%)	62 (30.4%)
Yes	7 (63.6%)	3 (75.0%)	7 (58.3%)	7 (77.8%)	34 (79.1%)	14 (77.8%)	4 (36.4%)	8 (88.9%)	28 (57.1%)	30 (78.9%)	142 (69.6%)

Key to table: fields not comprising any data are marked “-” instead of “0”. Pooled countries for analysis: East EU (partly in Asia): Russia, Serbia, Poland, North Macedonia, Hungary, Slovenia; North EU: Sweden, Netherlands, Denmark, Germany, Belgium; South EU: Italy, France, Spain, Portugal, Greece, Turkey; Africa: Egypt, Tunisia, South Africa; Singapore; Canada; United States of America; South America: Brazil, Argentina; United Kingdom; Australasia: Australia, New Zealand. **Involvement** in clinical research in SMA; **Interest** in conducting research in rehabilitation in SMA; **Opportunity** to publish in SMA.

HCP: Healthcare professional; DPT: Doctor of Physical Therapy; OTD: Doctor of Occupational Therapy.

The sample of respondents consists predominantly of physiotherapists,^30^ who made up 70.6% (144) of the participants. Other professional groups represented include speech and language therapists/speech language pathologist at 6.9% (14), occupational therapists at 9.3% (19), and respiratory therapists at 6.9% (14). Additionally, 6.4% (13) of the respondents are neuro and psychomotor therapists of developmental age (TNPEE), a specialized health care rehabilitation professional in Italy.^42^ An additional 20 individuals, who declared not to be actively engaged in SMA-related care or research, were excluded from the survey process and data were not used in the analysis.

**Figure 1.**
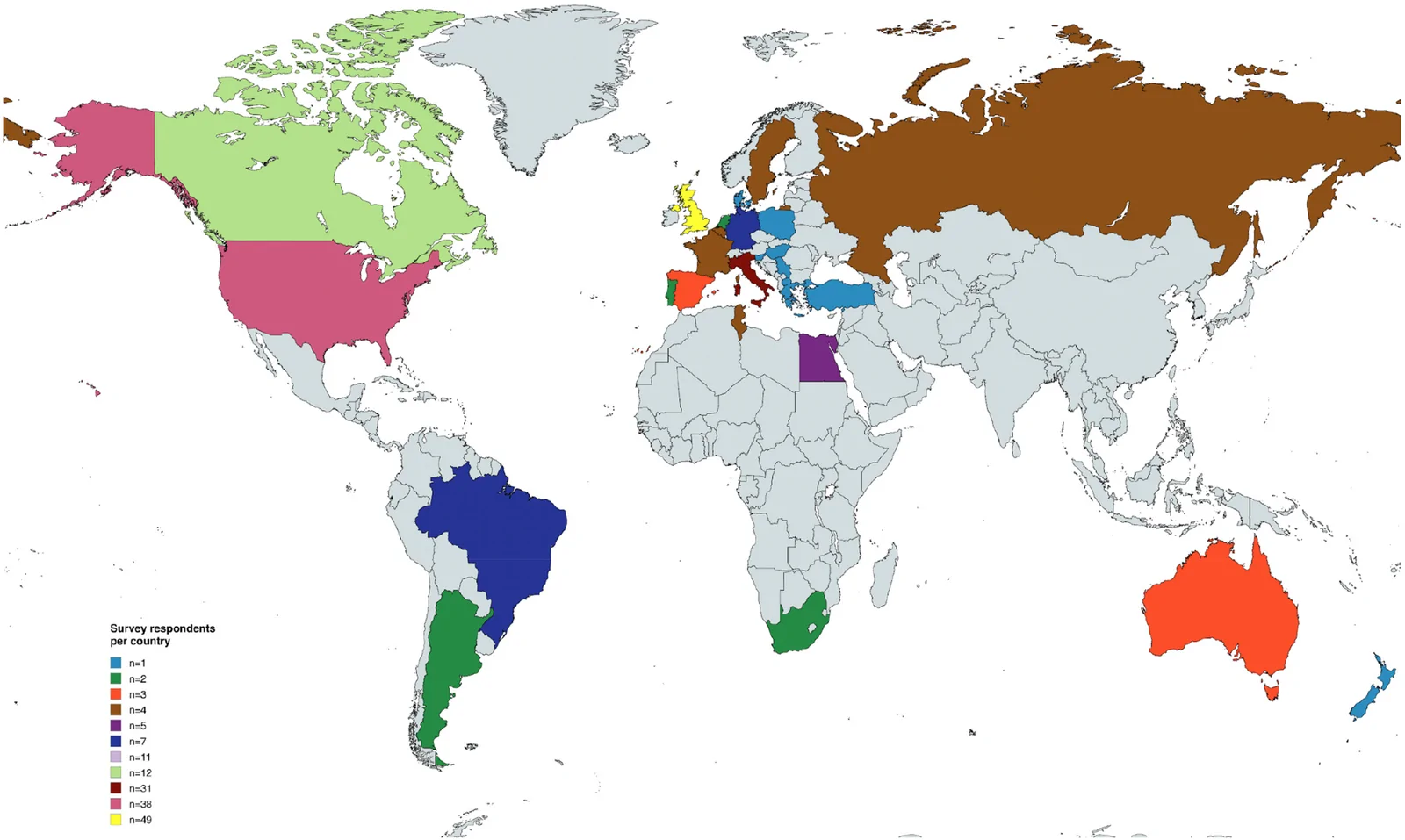
Number of survey respondents per country: Total = 204; n = 1 (Slovenia, Denmark, Serbia, Poland, North Macedonia, New Zealand, Hungary, Greece, Turkey); n = 2 (South Africa, Portugal, Netherlands, Argentina); n = 3 (Australia, Spain); n = 4 (Tunisia, Sweden, Russia, France, Belgium); n = 5 (Egypt); n = 7 (Germany, Brazil); n = 11 (Singapore); n = 12 (Canada); n = 31 (Italy); n = 38 (United States of America); n = 49 (United Kingdom).

Figure 1 displays a world map showing the number of respondents per country, highlighting the global distribution of participants involved in SMA care and research. Survey respondents were asked about their interest in conducting research in SMA and their current involvement in clinical research. The responses were cross tabulated to assess the relationship between interest in research and active involvement. Of the 26 respondents who indicated no interest in conducting rehabilitation research, the majority (84.6%, N = 22) were not involved in clinical research (pharma- and/or investigator-initiated clinical research), while 15.4% (N = 4) reported active participation in clinical research. Among the 178 respondents who expressed interest in conducting research, a significant proportion (74.2%, N = 132) were already involved in clinical research, while 25.8% (N = 46) were not involved.

The survey participants ranked nine potential research barriers based on their perceived importance.

### Analysis by demographic area

*Difficulty finding funding* was the top-ranked barrier in all regions, with mean ranks ranging from 1.67 (Australasia) to 3.21 (East Europe). *Difficulty accessing statistical or data management support* ranked second in most regions, including Canada (3.58), East Europe (4.12), Singapore (3.64), and the USA (3.5), but was lower in South Europe (#6, 6.39) and the UK (#5, 5.47).

*Lack of knowledge/capacity to design rehabilitation or trial protocols* ranked third in several areas (Canada #3, 4.00; USA #3, 4.79), but was lower in East Europe (#4, 5.25) and South America (#5, 5.39).

Other barriers varied more: *difficulty finding collaborators* was ranked second in Africa (4.02) and South Europe (4.27), but seventh in Canada (6.25). *Mentorship gaps* and *manuscript writing skills* ranked consistently lower. The least cited barriers were *difficulty engaging scientific interest* and *institutional constraints*, although the latter was ranked higher in North Europe (#3, 5.01) and the USA (#4, 5.11).

**Figure 2.**
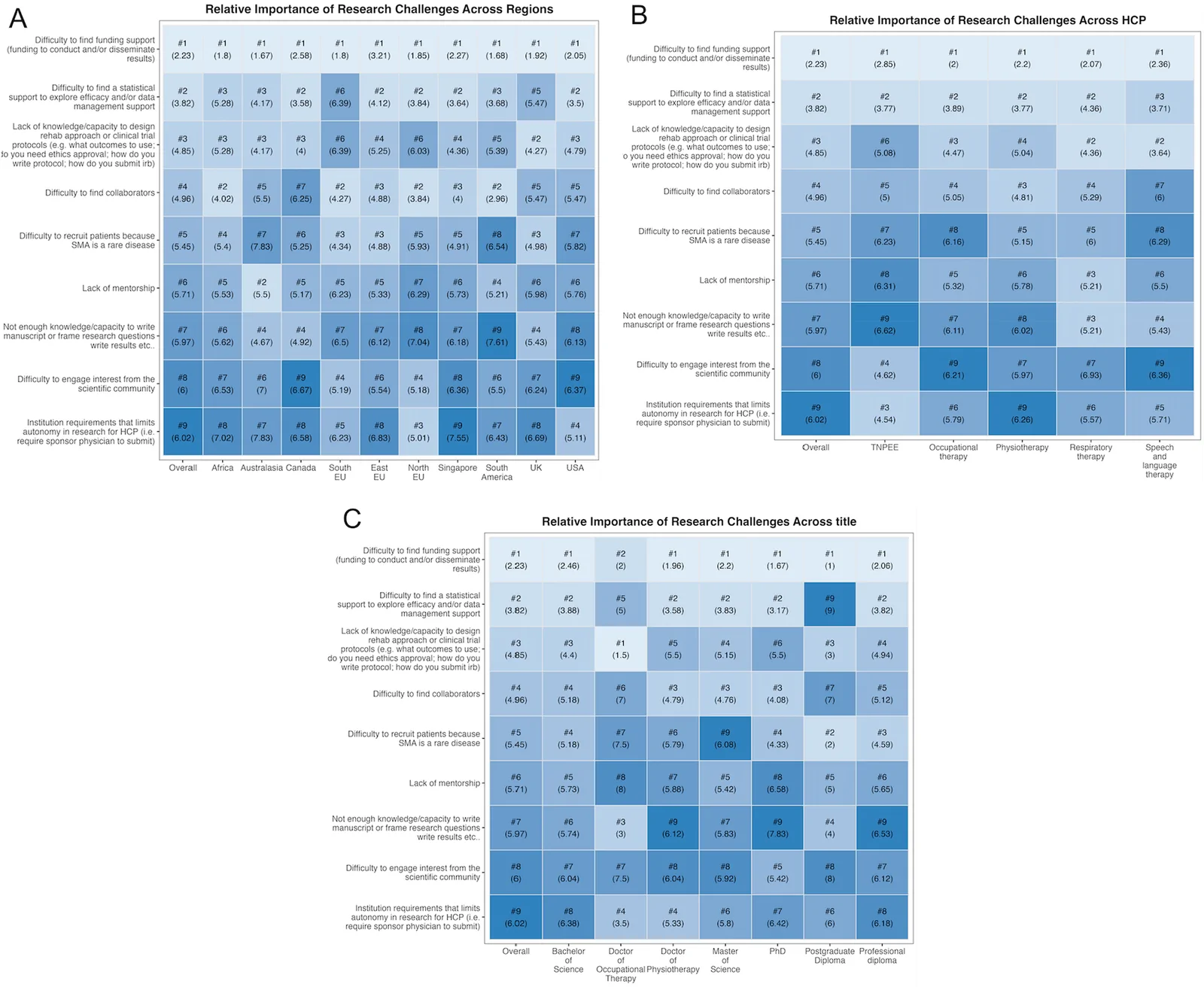
Ranked barriers to conducting rehabilitation research in SMA by demographic area, healthcare profession, education title.

The overall agreement among regional rankings was substantial, as indicated by Kendall's W = 0.65 (χ² = 52, p < 0.0001), demonstrating a statistically significant concordance across all regions analyzed (Figure 2A).

Pairwise Kendall's tau coefficients revealed varying degrees of association between individual regions. The highest correlation was observed between Africa and Singapore (τ = 0.944), which remained statistically significant after adjustment for multiple comparisons (adjusted p = 0.0022). Other regional pairs exhibited moderate to high tau values; however, these associations did not reach significance post-adjustment.

### Analysis by HCPs

All professional groups ranked *difficulty finding funding* as the top barrier (#1). *Statistical/data support* ranked second across most roles, except for speech and language therapy (#3). *Lack of capacity to design protocols* ranked #2–4 for most, but was #6 in TNPEE. *Finding collaborators* was deprioritized by speech and language therapists (#7).

*Mentorship* and *writing skills* ranked lower overall but were more important in respiratory and speech therapy. *Scientific engagement* and *institutional constraints* were least endorsed (#8–9), though ranked higher in TNPEE (#3–4).

Agreement across professions was moderate to strong (Kendall's W = 0.68; χ² = 27.6, p < 0.0001), indicating a statistically significant overall concordance in rankings. However, post hoc pairwise contrasts between individual professional groups did not reach statistical significance after adjustment for multiple comparisons. (Figure 2B)

### Analysis by education title

Across all education levels, *difficulty finding funding* was the top-ranked barrier (#1). *Statistical/data support* followed, except among Doctor of Occupational Therapy (#5) and Professional Diploma holders (#9). *Design capacity* was top-ranked by Doctor of Occupational Therapy and #3 among Diploma holders. Lower-ranked challenges included *mentorship*, *writing skills*, and *institutional constraints*, though some groups rated the latter higher (e.g., #4 for Doctor of Occupational Therapy).

There was moderate to strong agreement across education levels (Kendall's W = 0.68; χ² = 30.24, p < 0.0001)), indicating a statistically significant overall concordance in rankings. However, post hoc pairwise contrasts between individual education title groups did not reach statistical significance after adjustment for multiple comparisons. (Figure 2C)

**Figure 3.**
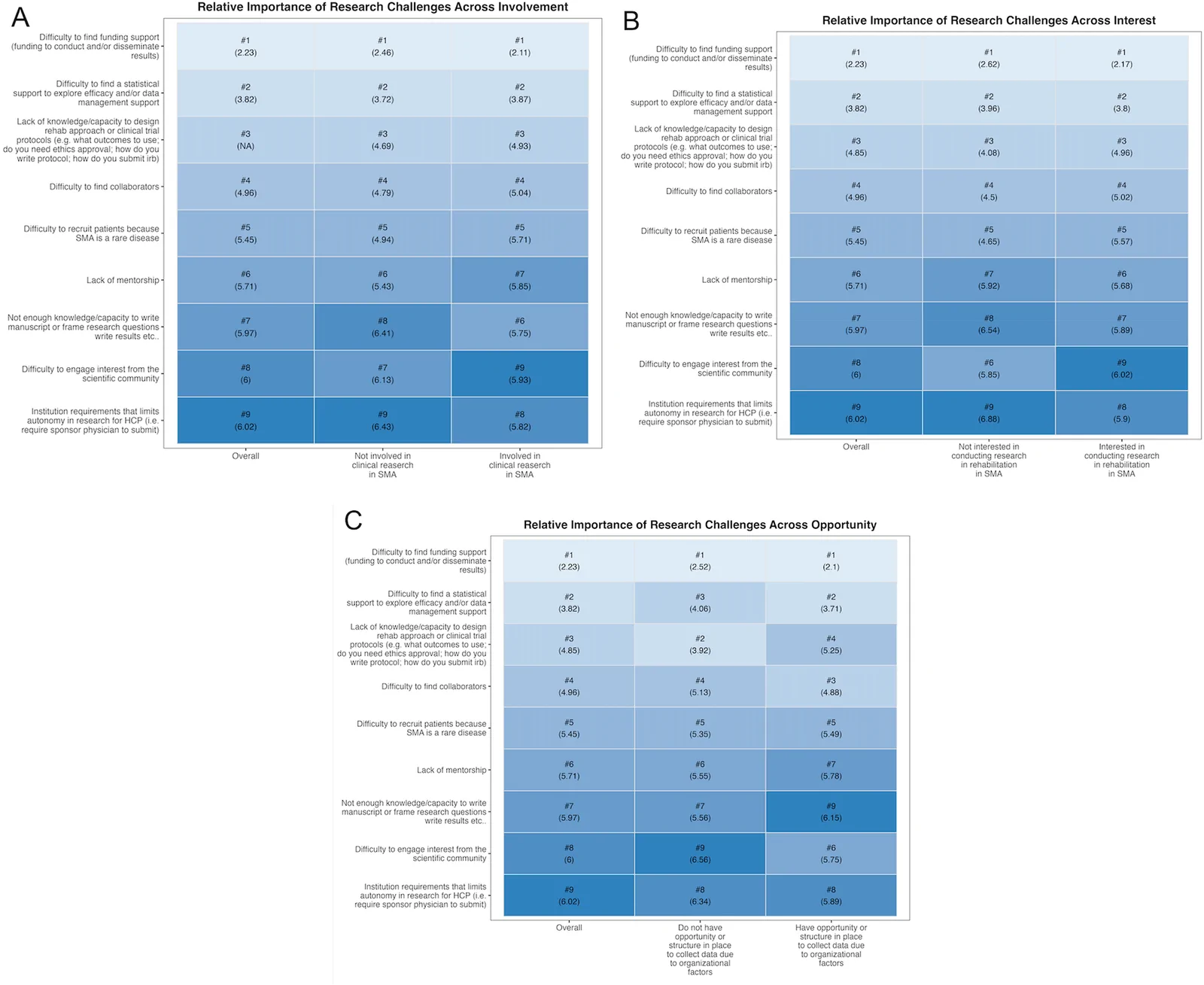
Ranked barriers to conducting rehabilitation research in SMA by research involvement, interest, and publishing opportunity.

As clinicians and clinician researchers have differing opportunities for research, findings were also subdivided by involvement, interest and opportunity of the professional in SMA clinical research (Figure 3).

Among those *involved and not involved in SMA clinical research*, difficulty securing funding was the top-ranked challenge, followed by difficulty accessing statistical or data management support and lack of capacity to design protocols. Lower-ranked barriers included mentorship gaps, manuscript writing skills, and institutional constraints. Individuals not involved in research rated manuscript writing and institutional limitations slightly higher. Agreement between the two groups was assessed using Kendall's tau correlation coefficient (τ = 0.78, p = 0.0024), indicating strong concordance. (Figure 3A).

Among those *interested and not interested* in conducting rehabilitation research in SMA, difficulty securing funding was ranked as the most significant barrier, followed by limited access to statistical/data support and limited capacity to design rehabilitation or trial protocols. Agreement between the two groups was assessed using Kendall's tau correlation coefficient (τ = 0.83, p = 0.008), indicating strong concordance. (Figure 3B).

Among those *with and without the opportunity to publish* difficulty securing funding was ranked as the most significant barrier (No opportunity: 2.52; Opportunity: 2.10), followed by *difficulty accessing statistical/data support* (No opportunity: 4.06; Opportunity: 3.71) and *lack of knowledge/capacity to design rehabilitation protocols or clinical trials* (No opportunity: 3.92; Opportunity: 5.25). Those without publication opportunity ranked protocol design higher (2nd vs 4th) and scientific engagement lower (9th vs 6th). Agreement between the two groups was assessed using Kendall's tau correlation coefficient (τ = 0.63, p = 0.013), indicating moderate to strong concordance (Figure 3C).

Based on responses to the open-ended question, several other recurring barriers such as time constraints, lack of organizational support, personnel shortages, limited resources, logistical challenges and lack of training and mentorship for conducting research were identified.

## Discussion

### Phase 1: literature review and quality assessment

The literature review results revealed a limited number of studies focused on evaluating the impact of rehabilitation interventions in the care of individuals with SMA.

Overall, the methodological quality of the reviewed studies was heterogeneous, with interventional studies generally demonstrating a higher prevalence of lower-quality evidence.

Common limitations included small sample sizes, lack of randomization, absence of control group, and inadequate reporting on methods of the intervention including adherence to interventions. Additionally, many studies suffered from short durations with poor to absent follow-up, preventing robust conclusions on long-term efficacy and the sustainability of implemented care strategies. Although these limitations are also inherent to research in rare disease, an effort should be made to overcome these barriers.

Interventional studies physiotherapy/physical therapy often rely on clinical outcome measures to assess efficacy or study disease trajectories, as they are readily accessible to both care and research teams.^43^ However, these measures typically require extended follow-up periods of 1–2 years to detect meaningful changes over time or does not directly relate to the mechanism of intervention.^44–46^ The benefits linked to physiotherapy/physical therapy are usually reflected within the quality of life and are the fruit of long-term practice.^47^ A combination of performance-based and observer-rated outcome measures with patient-reported outcomes applied in long-term studies could better inform the observed results.^48^

The current literature holds promise for the potential of bulbar targeted therapeutic effects. One key domain that has risen to importance post DMT era, is in feeding and swallowing, where deficits are often profound and limited treatments capable of making clinically significant improvements in the presence of profound impairments exist. Continuation of these research lines into evidence-based research could be of help to inform clinical care management.

In respiratory therapy, there was a high proportion of papers that did not meet minimal APTA CAT-EI criteria. This is likely due to the low number of patients reviewed, often case reports, coupled with minimal longitudinal follow up. Respiratory therapy in this area is a developing field, with the importance of specific research only coming to light since the introduction of DMTs. Although the involvement of respiratory therapists and/or pulmonologists in SMA care increased considerably in the last years, no interventional rehabilitation research has been published since 2022. It is an area which needs further focus with specific measures over longer timeframes.

The eligible studies related to occupational therapy do show a positive trend toward using various assistive devices but demonstrate the challenge of adapting the interventions for those with SMA in a real-life setting. However, there are not any currently available studies evaluating day to day function, social participation, occupational performance of people with SMA or the use of low-tech occupational therapy interventions with individuals with SMA.

Future research with larger SMA-specific cohorts, more robust designs, and longitudinal follow-up will be crucial for developing evidence-based rehabilitation therapy interventions tailored to the unique heterogeneous needs of individuals with SMA. Given the current challenges encountered in rehabilitation interventional studies, the availability of only positive trends on small samples found in studies classified with a lower quality should not constitute a barrier for its application in a real-world data collection setting.^23^

### Phase 2: the survey

To better understand these challenges faced in advancing research within the field of SMA rehabilitation, we conducted a global survey asking rehabilitation professionals around the world to identify and classify by order of importance the barriers encountered when trying to contribute to the field. Regardless of the HCP domain, respondents agreed on key barriers to conducting such studies. Difficulty obtaining funding (Rank 1), statistical support to explore efficacy and/or data management (Rank 2) and limited knowledge or capacity to design rehabilitation protocols or clinical trials (Rank 3) emerge as the most significant obstacles. Interestingly, while scientific community engagement appears to be a lesser barrier (Rank 8), the inclusion of SMA patients and the challenge of finding collaborators rank mid-range. Indeed, designing international research studies usually requires a robust network. A recurrent barrier added by the respondents was research time allocation. Indeed, many clinicians often do not have dedicated research time because of reduced personnel and lack of translational care-research structure within their institutions. This challenge persisted even at the country level, underscoring its universal impact.

### A call to action

With the exciting advancements in DMTs a paradigm shift is needed in how we approach the separation of clinical practice and research. We must learn from every patient in the clinic, especially in rare diseases like SMA because it not only improves research evidence but also improves quality of clinical care.^49^ Collaborative efforts among clinician researchers will improve translational research and knowledge within the field. Addressing these barriers require systemic changes, such as implementing institutional policies that allocate protected research time for data collection and analysis, support for attendance at international conferences to enable networking and providing infrastructure support to seamlessly integrate clinical interventions captured in a manner that may be publishable to improve rehabilitation knowledge for SMA.

To overcome these challenges, several strategies could be implemented. Integrating methodological and research training into rehabilitation education programs could provide the foundation necessary for research design and publication to advance research in their field throughout their careers. Fostering international collaboration among HCPs could address several barriers simultaneously. Collaborative efforts can strengthen funding proposals, enabling larger sample sizes and ensuring adequate resources for long-term studies. These partnerships could also facilitate access to multidisciplinary expertise, including statistical and methodological support, through mentorship programs or preceptorships with the aim of conducting clinical research to improve patient care. Furthermore, the involvement in study design and results dissemination of patient advocacy groups to represent patients’ voices; industry partners to support SoC improvement alongside DMT; and rehabilitation device companies to open new rehabilitation opportunities could play a pivotal role in HCP research focused on SMA.^50^ Current general grant programs often prioritize basic science or drug development, leaving rehabilitation research underfunded.^51^ This imbalance can make it difficult for rehabilitation-focused projects, as rehabilitation requires more engagement and adherence by the patient thus requiring different study designs, longer follow-up periods, and multidisciplinary collaboration that may not align with traditional study designs. Dedicated funding streams for rehabilitation research could help bridge this gap, ensuring that these crucial studies complement the current treatment landscape in SMA.

Reducing costs associated with staff involvement could also help in overcoming several problems and this might be achieved through the integration of wearable technologies. Although these devices are considered as costly, wearable technology is currently being studied to understand their potential to assist with telehealth/real world rehabilitation programs and detect earlier change on smaller samples compared to traditional clinical outcome measures.^47,52–56^

Several limitations have been identified. Notably, although all studies were published after 2016 and despite the transformative impact of DMTs since their introduction, a considerable proportion of studies were conducted in DMT-naive populations. Despite this changing landscape, most individuals with SMA have chronic SMA requiring a multidisciplinary approach to improve overall health outcomes. For the many who are not identified pre-symptomatically or have chronic SMA, a combination approach of medical treatment and rehabilitation would be required to optimize health outcomes with DMTs. Pre- vs post-symptomatic treatment for patients with SMA will require different approaches to rehabilitation. In addition, a general limitation of the review is that our search strategy may not have fully captured all relevant literature, particularly gray literature such as conference proceedings, posters, and other non-indexed sources. The survey participants could not represent a homogenous sample across all continents and countries, which may limit the generalizability of the findings. Indeed, since the survey was distributed through networks, only known HCPs involved in SMA were approached.

Yet, we could conclude that less experienced sites are unlikely to have the patient experience to expand on these barriers and are likely to experience the same issues as the experienced sites. The relatively small number of clinicians and researchers across the different HCP domains involved in SMA leads to underrepresentation of some domains. However, with the changing phenotype, other rehabilitation professionals’ expertise will be required beyond the previously known motor deficits.

Finally, it is likely that research barriers were underreported given the survey's use of a dichotomous (yes/no) response option for questions that may have involved complex or nuanced issues. This limitation could have restricted respondents’ ability to fully express the variety and depth of challenges they face. Nevertheless, the authors intentionally designed the survey to be as inclusive and accessible as possible, aiming to accommodate a broad spectrum of HCPs across diverse roles, settings, and levels of research experience.

In conclusion, this paper reinforces the need for resources and value to be placed on translational medicine to improve the impact of rehabilitation research and patient care in SMA. These findings underscore the urgent need for well-designed, high-quality and multicentric international studies to address these gaps. Improved evidence-based literature will impact future SoC guidelines based on this new era of DMTs and possible design of new validated outcome measures to address the deficits arising in treated individuals with SMA. Addressing the gaps in research implementation and clinical presentation of this new phenotype stimulate the development of new rehabilitation and care techniques adapted to each single healthcare system and clinical trials to improve health outcomes in patients with SMA and support their healthcare journey.

## Clinical message

- Lack of robust, evidence-based interventional rehabilitation research in SMA
- Narrative review permits to understand current rehabilitation research
- Survey helps to understand barriers faced by professionals to conduct research
- Mitigation strategies provided to increase interventional rehabilitation research

## Supplemental Material

sj-docx-1-jnd-10.1177_22143602251364945 - Supplemental material for Rehabilitation research in spinal muscular atrophy: a call to action Supplemental material, sj-docx-1-jnd-10.1177_22143602251364945 for Rehabilitation research in spinal muscular atrophy: a call to action by Charlotte Lilien, Leslie Nelson, Lisa Edel, Danielle Forrest, Timothy Estilow, Katlyn E McGrattan, Tina Duong and Giorgia Coratti in Journal of Neuromuscular Diseases

sj-docx-2-jnd-10.1177_22143602251364945 - Supplemental material for Rehabilitation research in spinal muscular atrophy: a call to action Supplemental material, sj-docx-2-jnd-10.1177_22143602251364945 for Rehabilitation research in spinal muscular atrophy: a call to action by Charlotte Lilien, Leslie Nelson, Lisa Edel, Danielle Forrest, Timothy Estilow, Katlyn E McGrattan, Tina Duong and Giorgia Coratti in Journal of Neuromuscular Diseases

sj-docx-3-jnd-10.1177_22143602251364945 - Supplemental material for Rehabilitation research in spinal muscular atrophy: a call to action Supplemental material, sj-docx-3-jnd-10.1177_22143602251364945 for Rehabilitation research in spinal muscular atrophy: a call to action by Charlotte Lilien, Leslie Nelson, Lisa Edel, Danielle Forrest, Timothy Estilow, Katlyn E McGrattan, Tina Duong and Giorgia Coratti in Journal of Neuromuscular Diseases

sj-docx-4-jnd-10.1177_22143602251364945 - Supplemental material for Rehabilitation research in spinal muscular atrophy: a call to action Supplemental material, sj-docx-4-jnd-10.1177_22143602251364945 for Rehabilitation research in spinal muscular atrophy: a call to action by Charlotte Lilien, Leslie Nelson, Lisa Edel, Danielle Forrest, Timothy Estilow, Katlyn E McGrattan, Tina Duong and Giorgia Coratti in Journal of Neuromuscular Diseases

sj-docx-5-jnd-10.1177_22143602251364945 - Supplemental material for Rehabilitation research in spinal muscular atrophy: a call to action Supplemental material, sj-docx-5-jnd-10.1177_22143602251364945 for Rehabilitation research in spinal muscular atrophy: a call to action by Charlotte Lilien, Leslie Nelson, Lisa Edel, Danielle Forrest, Timothy Estilow, Katlyn E McGrattan, Tina Duong and Giorgia Coratti in Journal of Neuromuscular Diseases

## Abbreviations (alphabetical order)

DMT: Disease-modifying therapy
HCP: Healthcare professional
NBS: Newborn screening
OT: Occupational therapists
PT: Physiotherapist/Physical therapist
SALT/SLP: Speech and language therapists/Speech language pathologist
SMA: Spinal muscular atrophy
SoC: Standard of care
