# Supplementary material for: Rehabilitation research in spinal muscular atrophy: a call to action
Source: J Neuromuscul Dis. 2025 Aug 25;13(3):232–47. doi: 10.1177/22143602251364945 (PMC13438077; doi:10.1177/22143602251364945)
Supplement: Supplementary file 1 [file sj-docx-1-jnd-10.1177_22143602251364945.docx]

**Supplementary 1**

To identify peer-reviewed publications relevant to different rehabilitation domains in Spinal Muscular Atrophy (SMA), a structured search was conducted using PubMed. A combination of Medical Subject Headings (MeSH) terms and keyword-based title or abstract searches was applied to capture the breadth of the literature.

**Search Strategy for Physiotherapy/Physical Therapy Literature Review in SMA**

Below is a description of the terms used:

- **(atrophy, spinal muscular[MeSH Terms]) AND (rehabilitation[Title])**
  This search combined the MeSH term for SMA with the keyword “rehabilitation” specifically in the article title, aiming to capture studies that focused explicitly on rehabilitation approaches in SMA.
- **(atrophy, spinal muscular[MeSH Terms]) AND (physiotherapy[Title])**
  This term pairing was intended to identify articles where “physiotherapy” appeared in the title, alongside the MeSH term for SMA. It reflects a common professional term used internationally.
- **(atrophy, spinal muscular[MeSH Terms]) AND (exercise[Title])**
  Used to retrieve studies emphasizing “exercise” as a primary focus in SMA rehabilitation.
- **(atrophy, spinal muscular[MeSH Terms]) AND ("Exercise"[Majr:NoExp])**
  Focused on studies where “Exercise” is a major topic, but without including all its narrower subcategories (i.e., No Explosion).
- **"spinal muscular atrophy" AND "physiotherapy[Title]"**
  A keyword-based search combining the phrase “spinal muscular atrophy” with “physiotherapy” in the title.
- **"spinal muscular atrophy" AND "rehabilitation[Title]"**
  Similar to above, but using “rehabilitation” as the targeted term in the article title.
- **"Spinal Muscular Atrophy" AND "Physical Therapy[Title]"**
  Captured articles using “physical therapy” in the title, which is an alternative to “physiotherapy” commonly used in U.S.-based literature.
- **"Spinal Muscular Atrophy" AND "Aquatic[Title]"**
  Designed to identify studies on aquatic therapy or pool-based interventions where “aquatic” appears in the title.
- **"Spinal Muscular Atrophy" AND "Aquatic[Title/Abstract]"**
  Broadened the previous search to include “aquatic” in either the title or abstract.
- **"Spinal Muscular Atrophy" AND "hydro[Title/Abstract]"**
  Searched for hydrotherapy-related studies by targeting the stem “hydro” in title or abstract fields.
- **"Exercise"[mesh] AND "spinal muscular atrophy" NOT "SBMA"**
  Used the MeSH term “Exercise” combined with SMA, excluding SBMA (Spinobulbar Muscular Atrophy) to maintain focus on classical SMA types.
- **("spinal muscular atrophy") AND (training[Title]) NOT ("SBMA")**
  Looked for studies with “training” in the title, excluding SBMA.
- **"Muscular Atrophy, Spinal/rehabilitation"[MAJR] NOT bulbar NOT Hirayama**
  Searched for articles where rehabilitation is a major topic in SMA, excluding conditions such as bulbar SMA and Hirayama disease to narrow the focus.

**Search Strategy for Respiratory Therapy Literature Review in SMA**

The search terms and their rationale are detailed below:

- **("Breathing Exercises"[Mesh]) AND "Muscular Atrophy, Spinal"[Mesh]**
  This combination retrieved studies indexed under both MeSH terms “Breathing Exercises” and “Muscular Atrophy, Spinal.” It targeted literature describing formal breathing exercise interventions in SMA populations.
- **("Respiration"[MeSH]) AND "Muscular Atrophy, Spinal"[Mesh] AND "rehabilitation"**
  Focused on studies exploring the intersection of respiratory function, SMA, and rehabilitation. This search captured publications discussing respiratory involvement and strategies to support breathing in a rehabilitative context.
- **("Respiratory Therapy"[Mesh]) AND "Muscular Atrophy, Spinal"[Mesh]**
  Used the MeSH terms for both “Respiratory Therapy” and “Spinal Muscular Atrophy” to identify literature specifically indexed under these concepts. This is a comprehensive approach to retrieving relevant articles on respiratory interventions tailored for SMA.
- **"airway clearance" AND "spinal muscular atrophy"**
  This keyword-based search targeted studies where airway clearance techniques (e.g., cough assist, suctioning, postural drainage) are mentioned in the context of SMA. It was included to capture clinically relevant techniques that may not always be indexed under formal MeSH terms.

**Search Strategy for Speech and Language Therapy (SALT) Literature in SMA**

The search terms and their rationale are detailed below:

- **(("Range of Motion, Articular"[Mesh]) AND "Muscular Atrophy, Spinal"[Mesh]) NOT "Bulbo-Spinal Atrophy, X-Linked"[Mesh] NOT "Amyotrophy, monomelic"[Supplementary Concept])**
  This search identified studies related to joint range of motion in SMA, capturing aspects of physical therapy intersecting with bulbar and oral-motor functions, while excluding related motor neuron disorders.
- **(("bulbar"[Title]) AND "Muscular Atrophy, Spinal"[Mesh]) NOT "Bulbo-Spinal Atrophy, X-Linked"[Mesh] NOT "Amyotrophy, monomelic"[Supplementary Concept])**
  This targeted literature with a focus on bulbar symptoms and involvement in SMA, crucial for understanding speech, swallowing, and respiratory challenges.
- **(("Deglutition Disorders/rehabilitation"[Mesh] OR "Deglutition Disorders/therapy"[Mesh]) AND "Muscular Atrophy, Spinal"[Mesh]) NOT "SBMA"**
  This search specifically retrieved publications addressing rehabilitation or therapy of swallowing disorders (dysphagia) in SMA, excluding Spinal Bulbar Muscular Atrophy (SBMA).
- **("Dysphagia"[Title/Abstract] AND "Muscular Atrophy, Spinal"[Mesh]) NOT "SBMA"**
  Focuses on literature discussing swallowing difficulties (dysphagia) in SMA patients, providing insights into clinical management and therapeutic approaches.
- **("Swallowing"[Title/Abstract] AND "Muscular Atrophy, Spinal"[Mesh]) NOT "SBMA"**
  Captures studies centered on swallowing function and related interventions in SMA, further informing speech and language therapy practices.

**Search Strategy for Occupational Therapy (OT) Literature in SMA**

The search terms and their rationale are detailed below:

- **"Occupational Therapy"[Mesh] AND "Muscular Atrophy, Spinal"[Mesh]**
  Targeted literature specifically indexed under occupational therapy and spinal muscular atrophy.
- **"Muscular Atrophy, Spinal/rehabilitation"[Majr] NOT bulbar NOT Hirayama**
  Captured articles focusing on rehabilitation in SMA excluding bulbar-related and Hirayama disease cases, thus including interventions potentially relevant to occupational therapy.
- **"assistive technology"[Title/Abstract] AND "spinal muscular atrophy"**
  Focused on studies discussing assistive technology and devices to support SMA patients, an important aspect of occupational therapy practice.
