# Supplementary material for: Rehabilitation research in spinal muscular atrophy: a call to action
Source: J Neuromuscul Dis. 2025 Aug 25;13(3):232–47. doi: 10.1177/22143602251364945 (PMC13438077; doi:10.1177/22143602251364945)
Supplement: Supplementary file 3 [file sj-docx-3-jnd-10.1177_22143602251364945.docx]

**Supplementary 3: The survey**

Background:

In spinal muscular atrophy (SMA), disease-modifying therapies (DMT) alongside early treatment and proactive care as highlighted by the standard of care (SoC) changed the disease landscape of SMA, leading to new challenges for clinical care teams. In addition, the quantitative and qualitative disparity in care provision remains universal, forcing healthcare professionals (HCPs) in SMA to redefine the care guidelines.

The availability of disease-modifying treatments underscores the necessity of combining them with rehabilitation. However, this also highlights the lack of quality studies to ascertain the effectiveness of rehabilitation treatments. The implementation of rehabilitation in care requires randomized controlled trials to enable high-quality evidence-based research and provide robust results, which is particularly lacking in the neuromuscular field.

This research project aims to identify and elaborate on the potential barriers and limitations in HCP research in SMA with a focus on domains in rehabilitation medicine: physiotherapy, occupational therapy, speech and language therapy and respiratory.

Aim of the survey:

Based on a literature review and our own experience, certain barriers across the various domains have been identified. With this survey, we aim to grasp the impact that these barriers might pose for you as an HCP in SMA when you seek to conduct research in your field and translate published standards of care into your care management.

What we will ask from you:

You have been invited to complete this survey because you are an HCP and involved in a research or clinical centre including patients with SMA.

This survey takes approximately 10 minutes and is free, without any obligation. It will be anonymous but we will require some professional experience information as well as your country.

While there is no remuneration available, you can specify in the survey if you wish to be acknowledged in the paper.

Do you want to participate at the survey? *Yes	No

Are you actively taking care of SMA patients (clinical care and/or research)? *Yes	No

Please select your country of practice *

Choose your HCP rehabilitation domain *

Physiotherapy rehabilitation, Occupational therapy rehabilitation, Speech and language therapy rehabilitation, For italians only: TNPEE, Respiratory therapy rehabilitation

Select your highest qualification*

Professional diploma (no university studies are needed in my country to practice my profession), Bachelor of Science (BsC), Master of Science (MsC), Doctor of Physiotherapy (DPT), Doctor of Occupational Therapy (OTD), Other, PhD

If you have selected "Other" please tell us which one *

Are you involved in clinical research in SMA? *Yes	No

Do you have interest in conducting research in rehabilitation in SMA in your HCP rehabilitation domain? *Yes	No

Do you have the opportunity or structure in place to collect data in a way that enables you to publish your findings within your rehabilitation domain as an HCP in your center? (due to organizational factors, your role, etc.) *Yes	No

Is there any other reason we missed in the previous question?

Do you wish to be acknowledged in the article *Yes	No

Please give us your details so we can acknowledge you in the article *

Name, First name
