# Supplementary material for: Rehabilitation research in spinal muscular atrophy: a call to action
Source: J Neuromuscul Dis. 2025 Aug 25;13(3):232–47. doi: 10.1177/22143602251364945 (PMC13438077; doi:10.1177/22143602251364945)
Supplement: Supplementary file 4 [file sj-docx-4-jnd-10.1177_22143602251364945.docx]

Supplementary 4: Quality assessment: APTA Critical Appraisal Tool for Experimental Intervention Studies: B. Quality Evaluation-Overall Design.

| Domain | Author, year | B1: inclusion criteria | B2: exclusion criteria | B3: recruitment methods attempt to minimize bias in sample | B4: a priori power analysis to determine sample size | B5: randomly assigned to intervention groups | B6: concealed allocation to treatment group | B7: control group | B8: subjects blinded to treatment group | B9: interventions described to be reproducible | B10: adherence described | B11: presence or absence of Adverse events described | B12: conflicts of interests managed to avoid biased results | Total score: yes=1; no=0 |
| --- | --- | --- | --- | --- | --- | --- | --- | --- | --- | --- | --- | --- | --- | --- |
| Motor rehabilitation | Gamze Bora, 2018 | yes | yes | no | no | no | no | no | no | yes | yes | yes | yes | 6 |
|  | Habets, 2022 | yes | yes | yes | no | no | no | yes | no | yes | yes | yes | yes | 7 |
|  | Immarino, 2024 | yes | yes | no | no | no | no | no | no | yes | yes | yes | yes | 6 |
|  | Nishikawa, 2021 | NA | NA | NA | NA | NA | NA | yes | no | yes | yes | yes | yes | 5 |
|  | Bulut, 2019 | NA | NA | NA | NA | NA | NA | no | no | yes | yes | yes | yes | 4 |
|  | Stark, 2018 | yes | yes | yes | no | no | no | no | no | yes | yes | yes | no | 6 |
|  | Nakajima, 2021 | yes | yes | yes | no | yes | yes | yes | yes | yes | yes | yes | yes | 11 |
|  | Gobbo, 2019 | NA | NA | NA | NA | NA | NA | no | no | yes | yes | yes | yes | 4 |
|  | Mirea, 2022 | yes | yes | yes | no | no | no | yes | no | no | yes | no | yes | 6 |
|  | Leung Yu, 2023 | yes | yes | no | no | no | no | no | no | yes | yes | yes | yes | 6 |
|  | Cumplido‑Trasmonte, 2022 | yes | yes | no | no | no | no | no | no | yes | yes | no | no | 4 |
|  | Moshonkina, 2024 | yes | yes | no | no | no | no | no | no | yes | yes | yes | no, | 5 |
|  | Lammers,2024 | no | no | no | no | no | no | no | no | yes | yes | yes | NA | 3 |
|  | Taylor, 2024 | NA | NA | NA | NA | NA | NA | no | no | yes | yes | no | no | 2 |
|  | Novikov, 2023 | no | no | no | no | no | no | no | no | yes | yes | yes | yes | 4 |
|  | Ahmed, 2024 | NA | NA | NA | NA | NA | NA | no | no | yes | yes | no | yes | 3 |
|  | Kanner, 2025 | NA | NA | NA | NA | NA | NA | no | no | yes | yes | yes | yes | 4 |
|  | Prat-Ortega, 2025 | yes | no | no | no | no | no | no | no | yes | yes | yes | yes | 5 |
| Respiratory care | Bertelli, 2017 | NA | NA | NA | NA | NA | NA | no | no | no | no | no | yes | 1 |
|  | Chen, 2016 | NA | NA | NA | NA | NA | NA | no | no | yes | yes | no | yes | 3 |
|  | Verrillo, 2019 | yes | yes | no | no | no | no | yes | no | yes | no | no | yes | 5 |
|  | Panitch , 2017 | no | no | no | no | no | no | no | no | no | no | no | no | 0 |
|  | Bach, 2017 | NA | NA | NA | NA | NA | NA | no | no | no | no | no | no | 0 |
|  | Senel, 2020 | yes | no | no | no | no | no | yes | no | no | no | no | no | 2 |
|  | Bach, 2019 | NA | NA | NA | NA | NA | NA | no | no | no | no | no | no | 0 |
|  | Suh, 2018 | no | no | no | no | no | no | no | no | no | no | no | no | 0 |
|  | Chatwin, 2020 | yes | yes | no | no | no | no | no | no | yes | yes | yes | yes | 6 |
|  | Veldhoen, 2022 | yes | yes | no | no | no | no | no | no | no | no | no | no | 2 |
| Occupational therapy | Rice et al, 2019 | yes | yes | no | no | no | no | yes | no | yes | yes | yes | no | 6 |
|  | Varela Aldas, et al 2024 | no | no | no | no | no | no | no | no | yes | yes | yes | yes | 4 |
|  | Vogel, et al 2018 | no | no | no | no | no | no | no | no | yes | yes | yes | yes | 4 |
|  | Janssen, 2021 | yes | yes | no | no | no | no | no | no | yes | yes | yes | no | 5 |
| Speech and language therapy | Chilińska-Pułkowska, 2021 | no | no | no | no | no | no | no | no | yes | yes | yes | no | 3 |
|  | Okamoto, 2024 | no | no | no | no | no | no | no | no | yes | yes | yes | yes | 4 |
|  | Morris, et al 2020 | no | no | no | no | no | no | no | no | yes | yes | yes | yes | 4 |
|  | McGrattan, et al 2025 | yes | yes | NA | no | no | no | no | no | yes | yes | no | NA | 4 |

Attribution of yes=1; no=0; NA (assigned for case studies/reports)=0 to estimate the overall quality of the study.
