# Supplementary material for: Rehabilitation research in spinal muscular atrophy: a call to action
Source: J Neuromuscul Dis. 2025 Aug 25;13(3):232–47. doi: 10.1177/22143602251364945 (PMC13438077; doi:10.1177/22143602251364945)
Supplement: Supplementary file 6 [file sj-docx-2-jnd-10.1177_22143602251364945.docx]

**Supplementary 2**
